# Thermally tunable electromagnetic surface waves supported by graphene loaded indium antimonide (InSb) interface

**DOI:** 10.1038/s41598-023-45475-8

**Published:** 2023-10-30

**Authors:** M. Z. Yaqoob, Munir Ahamd, A. Ghaffar, F. Razzaz, S. M. Saeed, T. M. Alanazi

**Affiliations:** 1 Department of Physics, Government College University, Faisalabad, 38000 Pakistan; 2 Department of Physics, University of Agriculture, Faisalabad, Pakistan; 3 Electrical Engineering Department, College of Engineering, Prince Sattam Bin Abdulaziz University, 16278 Al-Kharj, Saudi Arabia; 4 Faculty of Engineering and Information Technology, Taiz University, 6803 Taiz, Yemen

**Keywords:** Optics and photonics, Optical materials and structures, Optical techniques

## Abstract

The thermal agitation plays a vital role in tunability of optoelectronic, structural and chemical characteristics of the temperature sensitive materials. Graphene enables the THz optics, due to its unprecedent controlling characteristics over the traditional materials. The influence of temperature on the monolayer graphene is very negligible due to its low free charge carrier density, to enhance the thermal sensitivity of graphene, the graphene loaded temperature sensitive material interface has been proposed. A theoretical analysis has been carried out on temperature dependent propagation characteristics of electromagnetic surface waves supported by the graphene loaded semi-infinite indium antimonide (InSb). The InSb has been taken as temperature sensitive material. The Drude model has been used for the modeling of InSb in the THz region while the modeling of the graphene has been done by random phase approximation-based Kubo’s formulism. To realize the graphene loaded indium antimonide interface, the impedance boundary conditions (IBCs) have been employed. The numerical analysis has been conducted to analyze the influence of temperature on the characteristics of electromagnetic surface waves i.e., dispersion curve, effective mode index (N_eff_), penetration depth (δ), propagation length (L_p_), phase speed (V_p_) and field profile, propagating along the graphene loaded InSb. In all the numerical results, the temperature variation has been considered from 200 to 350 K. It has been concluded that the graphene–InSb interface provides more temperature assisted tunability to the interfacial surface modes, commonly known as surface waves, as compared to monolayer graphene. Further, the graphene parameters can play a vital role in the dynamical tuning of electromagnetic surface waves in THz to IR frequency range. The numerically computed results have potential applications in designing of thermo-optical waveguides, temperature assisted communication devices, thermo-optical sensors and near field thermal imaging platforms.

## Introduction

Electromagnetic surface waves have exciting applications in surface communication, optical sensing, chemical sensing, spectroscopy and near-field imaging^1–5^. The electromagnetic surface waves propagate along the interface of two dissimilar media and decays exponentially as moved away from the interface^6^. The different types of surface waves can be guided by interface of different partnering materials i.e., the metal–dielectric interface supports the surface plasmon polaritons (SPPs) wave, the dielectric-anisotropic dielectric interface supports the Dyakonov surface waves, the nonlinear and chiral interface guide the nonlinear and chiral surface waves respectively^7–9^. The characteristics of the surface waves highly depends upon the nature and characteristics of the partnering material^10^. The tunability of the surface waves is the major demand for their practical implementation. To overcome this issue, the different passive and active techniques have been developed to tune the surface waves^11^. Meanwhile, the different studies on corrugated semiconductors-based structures for plasmonic spoof have been conducted by different researchers to tune the propagation band of the SPP waves from visible range to THz range^12–15^.

The control over the characteristics of partnering materials plays vital role in the tuning and propagation of surface waves. Any physical or chemical sources near to the interface can induced the variations in the characteristics of surface waves, which provides the chemical sensing, optical sensing, defect sensing^16^. Similarly, an interface of partnering material can guide the surface wave at different temperature if the constitutive parameters of these materials are sensitive towards temperature. In literature, indium antimonide (Insb) and vanadium dioxide (VO_2_) have been extensively studied as temperature sensitive semiconductor materials due to their temperature dependent metal–insulator transition characteristics^17,18^. The temperature dependent electromagnetic surface waves supported by the temperature sensitive materials (TSMs) have been reported by many researchers i.e., Mackay and Lakhtakia solved the canonical boundary value problem based upon the uniaxial material and isotropic indium antimonide (InSb) material and reported the temperature dependent transition of Dyakonov waves to SPP waves at 0.6 THz frequency^19^. Further they extended their work and carried out the theoretical investigations on the temperature dependent hyperbolic materials composed of Insb and reported the propagation of surface waves with negative phase velocity at 2 THz frequency range^20^. In parallel, Fedorin numerically analyzed the influence of temperature on the surface waves supported by the planar interface between the porous nanocomposite material and hypercrystal composed of n-type InSb semiconductor layers and reported that the effective mode index, penetration depth and propagation frequency range are sensitive to the temperature. Moreover, a detailed analysis has been presented on the dissipation factors which affects the propagation of surface plasmon modes as well as hybrid additional modes and concluded that the under appropriate temperature range the propagation frequency band and surface wave parameters can be enhanced^21^. Recently, the theoretical investigation on the temperature dependent propagation of SPP waves supported by the silver/vanadium-dioxide interface has been carried out for the temperature sensing and crystallographic phase detection applications. The characteristics of SPP wave are found sensitive to the entire range of thermal hysteresis of VO_2_ i.e., that as the surface waves follows the thermal hysteresis^22^.

Until now, the temperature dependent electromagnetic surface waves supported by the InSb have been investigated for only some values of visible and near IR frequencies, however the extended propagation frequency range is major requirement for the broad applications. To full fill this gap, the graphene coated indium antimonide heterostructure has been proposed for temperature dependent surface waves for THz to IR board propagation band frequency range. The Indium Antimonide (InSb) is a small band gap semiconductor material which have potential applications in designing the thermal imaging cameras, FLIR systems, infrared homing missile guidance systems, and infrared astronomy^23^. Generally, the indium antimonide detectors are sensitive to the wavelength range 1–5 µm^24^.

Meanwhile, the graphene based plasmonic devices has open up new horizons for the researchers regarding the active control and manipulation of THz to IR band gap which are not possible by traditional liquid crystal and micromirror based devices. The unusual and extraordinary mechanical, electronic, optical and thermal properties of graphene made it suitable candidate for the plays important role in the m^25^. However, as the graphene is the allotrope of carbon, displays one atom-thick hexagonal configuration with low carrier density of free electrons, the efficiency of such devices is not so high as traditional devices. To enhance the optical, electrical and thermal properties of graphene, the different schemes as well as interfaces have been studied i.e., Lan et al., examined the transmission based highly efficient IR modulation assisted by the asymmetric light plasmon coupling on graphene nanoribbons based platforms and reported that the increase of 4% to 41% in the efficiency^26^. Zhao et al., worked on the electrochemical characteristics of graphene based composite organic structure (PMDA-NiPc-G) for charge transportation characteristic of the lithium ions batteries and reported that the incorporation of graphene improves the charge kinetics of lithium ions in the two dimensional grid structure^27^. Chen et al., investigated the structural, optical and electronic properties of the graphene–Bi_2_O_2_Se heterojunction under Van der Walls interaction in the frame work of density functional theory and reported that the control over the interfacial length between the graphene and Bi_2_O_2_Se can tune the photoelectric characteristics of the heterojunction^28^.

Du et al., achieved the thermal electrons via photoexcitation pumping of the mid IR pulses on the stacked layers of graphene via Bernal sequence and reported the observation of the near IR wavelengths of hot electrons. Moreover, the correlation between the number of staking layers and photocurrents has been determined and concluded that the photo-excited currents depend upon the number of staked layers, which leads to the electron thermalization process and ultrafast mid IR photodetectors^29^. In such studies, it is very important to understand the effect of temperature near to the interface of graphene and partnering materials and their interfacial plasmonic modes. To address this issue, the graphene loaded indium antimonide (InSb) interface has been studied for the temperature assisted plasmonic modes. The InSb has been chosen as temperature sensitive material because it has wide applications in IR as detectors, optoelectronics, microelectronics and solar cells and photovoltaics. Due to monolayer thickness and low charge density of the free electrons, the thermal response of the single layer graphene is very low and not sufficient for the efficient response to the thermal agitation. It is hypothesized that the thermal response of the graphene layer can be enhanced by the adding the TSM substrate. Keeping in view of these requirements, the theoretical study on the propagation of surface waves supported by the graphene loaded indium antimonide (InSb) interface has been conducted to achieve the subsequences objectives i.e., to enhance of the thermal sensitivity IR sensors/detectors based on the indium antimonide by graphene deposition, to enhance the thermal response of the monolayer graphene, and to get the wide propagation range of plasmon modes range from THz to IR region. For this purpose, the characteristic equations for TM-polarized and TE-polarized surface wave have been calculated analytically. The numerical results for the effective mode index (N_eff_), penetration depth (δ), propagation length (L_p_), phase velocity (V_p_), field profile and dispersion curve have been presented. The partition of the work is as follows i.e., “Analytical formulation” section presents the analytical methodology, the numerical results have been discussed in “Numerical results and discussion” section, while “Concluding remarks” section has the concluding remarks.

## Analytical formulation

**Figure 1:**
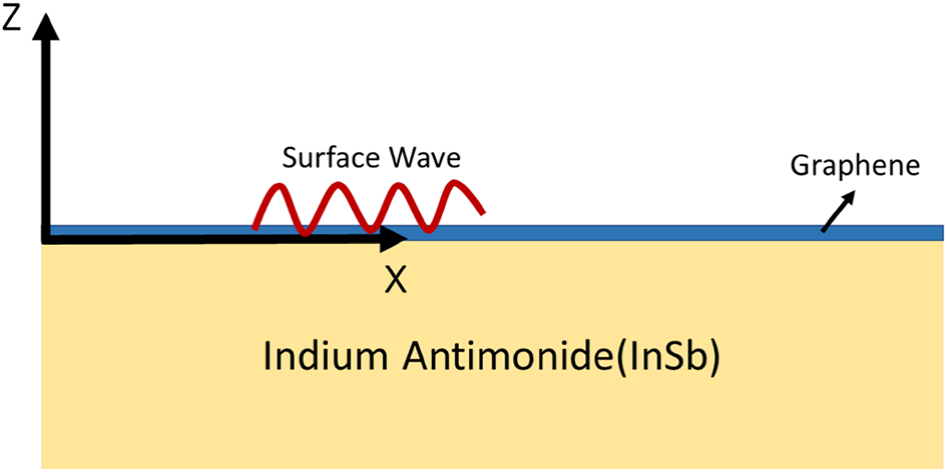
Geometry for propagation of electromagnetic surface waves supported by the graphene-coated indium antimonide.

The analytical formulation based upon the canonical boundary value problem for the propagation of surface waves supported by the graphene-loaded temperature sensitive material i.e., indium antimonide (InSb) has been presented in this section. The geometry of the problem is depicted in Fig. 1. To calibrate the electromagnetic characteristics of each medium, three divisions of the space have been made with respect to z-axis i.e., the region z < 0 is considered as indium antimonide (InSb) region, while the region z > 0 is occupied by free space and Graphene is considered as the one-atom thick single layer of carbon atom at z = 0.

The dispersive constitutive relations i.e., ε_2_(ω) and μ_2_(ω) have been used to model the TSM media, while the Kubo’s formulation is used in the analytical modeling of graphene. The optical conductivity (σ_g_) of the graphene layer is modeled as the function of operating frequency (ω), electron-photon scattering rate (τ), chemical potential (${\mu }_{c}$) and temperature (T). The explicit expression based on the random phase approximation is given as^30,31^

1 $${\sigma }_{g}\left(\omega ,{\mu }_{c},T,{\gamma }_{c}\right)=\frac{{e}^{2}\left(\omega +{\gamma }_{c} \right)}{i\pi {\hslash }^{2}}\left[\underset{-\infty }{\overset{+\infty }{\int }}\frac{\left|\in \right|}{(\omega +i{\gamma }_{c}{)}^{2} }\frac{d{f}_{0}\left(\varepsilon \right)}{d\varepsilon }-\int \limits_{0}^{+\infty }\frac{{f}_{0}\left(-\varepsilon \right)-{f}_{0}\left(\varepsilon \right)}{(\omega +i{\gamma }_{c}{)}^{2}-4\left(\frac{\varepsilon }{{h}^{2}}\right) }d\varepsilon \right],$$

where ‘e’ denotes the electron charge, ${\gamma }_{c}$ denotes a phenomenological carrier scattering rate which is energy independent. ${f}_{0}(\varepsilon )={\left({\text{exp}}^{\left(\frac{\varepsilon -{\mu }_{c}}{{k}_{B}T}\right)}+1\right)}^{-1}$ is the Fermi function, µc is the chemical potential (adjusted with a gate voltage), K_B_ is Boltzmann’s constant, ℏ is reduced Planck constant and T is the ambient temperature^30^. The first part in the equation refers to the graphene’s intraband conductivity caused by electron-photon scattering processes, whereas the second term refers to direct interband electron transitions. It should be highlighted that because the term µ_c_/K_B_T is smaller than 1 for doped and strongly gated voltages, the charge carrier density and chemical potential can be represented as n_s_ = µ_c_/π ℏ^2^ ${v}_{f}^{2}$ and µc $\cong \sqrt{\uppi {\mathrm{\hslash }}^{2}{v}_{f}^{2}}{n}_{s}$, respectively. The charge carrier density is very low in undoped (no chemical additions) and ungated (zero gate voltage) graphene at 70 K, and µ_c_/K_B_T is larger than 1, but it may be adjusted by chemical additions (doping) or with the assistance of a continuous electric field (electric field effect, gate voltage)^32^. The analytical formulation has been presented for the two states of polarization i.e., Transverse magnetic (TM) and secondly the transverse electric (TE) polarization. The Propagation of surface waves is supposed to be along the x-direction, as showed in Fig. 1.

### TM-polarized surface wave

Field equations for the transverse magnetic (TM) mode of polarization of surface waves, supported by Graphene-loaded temperature sensitive material for each region are as given in Refs.^6,21^;

for region z > 0,

2 $${H}_{y}=A{e}^{i\beta x}{e}^{-{k}_{1}z},$$

3 $${E}_{x}= - i\frac{{k}_{1}A}{\omega {\varepsilon }_{0}}{e}^{i\beta x}{e}^{-{k}_{1}z},$$

4 $${E}_{Z}=\frac{\beta A}{\omega {\varepsilon }_{0}}{e}^{i\beta x}{e}^{-{k}_{1}z},$$

where ${k}_{1}=\sqrt{{\beta }^{2}-{\omega }^{2}{\varepsilon }_{0}{\mu }_{0}}$ is attenuation constant.

For region z < 0

5 $${H}_{y}=B{e}^{i\beta x}{e}^{{k}_{2}z},$$

6 $${E}_{x} = \frac{i{k}_{2}B}{\omega {\varepsilon }_{2}}{e}^{i\beta x}{e}^{{k}_{2}z},$$

7 $${E}_{z}=\frac{\beta B}{\omega {\varepsilon }_{2}}{e}^{i\beta x}{e}^{{k}_{2}z},$$

where ${k}_{2}=\sqrt{{\beta }^{2}-{\omega }^{2}{\varepsilon }_{2}{\mu }_{2}}$ is attenuation constant, A and B are unknown coefficients, ${e}^{\pm {k}_{i}z}$ is exponentially decaying factor and ω is terahertz radiation frequency. The Impedance boundary conditions (IBCs) under the continuity of the tangential fields at the interface z = 0 are^33^

8 $${E}_{x}/{z}_{={0}^{+}}= {E}_{x}/{z}_{={0}^{-}},$$

9 $${H}_{y}/{z}_{={0}^{+}} - {H}_{y}/{z}_{={0}^{-}} = {\sigma }_{g}{E}_{x}/{z}_{=0}.$$

By incorporating Eqs. (2)–(7) in Eqs. (8) and (9), the following characteristics equation for TM-polarized surface wave has been computed,

10 $$\frac{{k}_{2}}{{k}_{1}}+\frac{{\varepsilon }_{2}}{{\varepsilon }_{1}}-\frac{ {k}_{2}{\sigma }_{g}}{i\omega {\varepsilon }_{o}}=0,$$

### TE-polarized surface wave

The mode of propagation of surface wave in which the electric field (E) is supposed to be in direction perpendicular to the plane of incidence, is called transverse electric (TE) polarized surface wave. The associated field phasors are given as^6,21^;

for region z > 0

11 $${E}_{y}=A{e}^{i\beta x}{e}^{-{k}_{1}z},$$

12 $${H}_{x}=i\frac{{k}_{1}A}{\omega {\mu }_{0}}{e}^{i\beta x}{e}^{-{k}_{1}z},$$

13 $${H}_{Z}=\frac{\beta A}{\omega {\mu }_{0}}{e}^{i\beta x}{e}^{-{k}_{1}z},$$

and for region z < 0,

14 $${E}_{y}=B{e}^{i\beta x}{e}^{{k}_{2}z},$$

15 $${H}_{x} = \frac{i{k}_{2}B}{\omega {\mu }_{1}}{e}^{i\beta x}{e}^{{k}_{2}z},$$

16 $${H}_{z}=\frac{\beta B}{{\omega \mu }_{1}}{e}^{i\beta x}{e}^{{k}_{2}z}.$$

The characteristic equation has been derived by after enforcing the impedance boundary conditions (IBCs) on the field phasors given in Eqs. (11)–(16) as^33^

17 $$\frac{{\mu }_{0}}{{\mu }_{1}} + \frac{{k}_{1}}{{k}_{2}}- {\sigma }_{g}\frac{i\omega {\mu }_{0}}{{k}_{2}}=0.$$

To get more insight into understanding the physical significance, the numerical results have been presented for these characteristic equations in the next section.

## Numerical results and discussion

In this section, the numerical results, regarding the electromagnetic surface wave propagating along the graphene-loaded indium antimonide (InSb) interface, have been presented. In the first part, the electromagnetic modeling of InSb and its temperature dependent insulator to metal transition characteristics has been analyzed under different temperature range. In second part, the possible numerical solution for electromagnetic surface waves supported by graphene–InSb interface has been computed. All these numerical computations have been executed in the Wolfram Mathematica software pack.

### Modeling of temperature-sensitive material (InSb)

**Figure 2:**
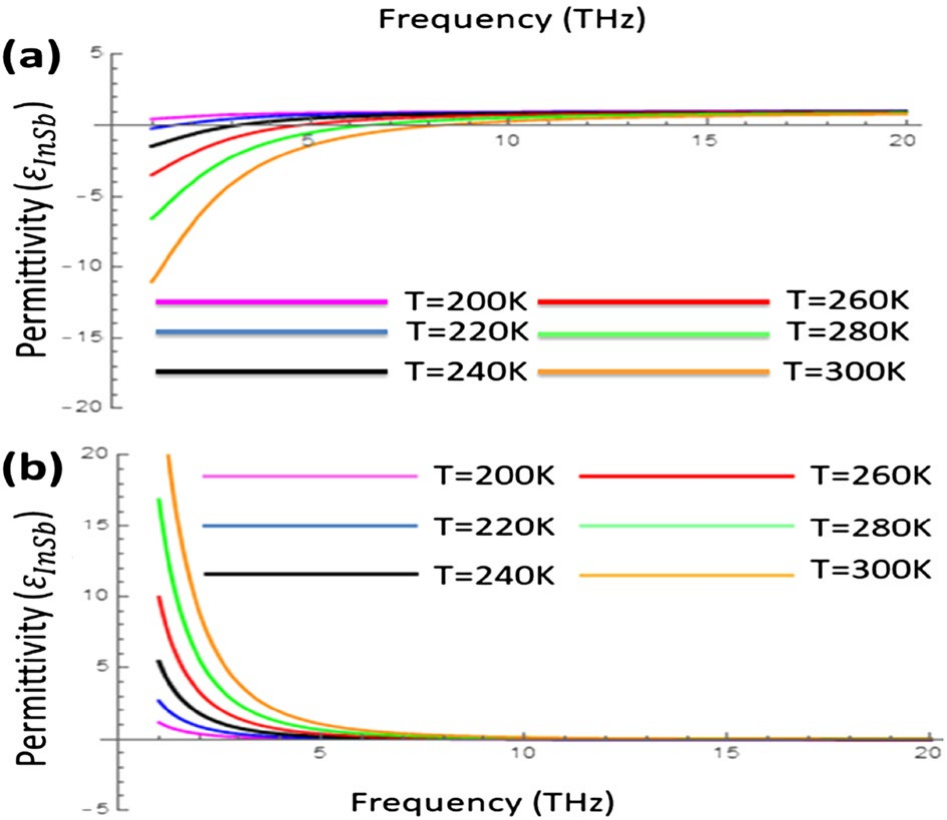
Relative permittivity of InSb (${\varepsilon }_{\text{InSb}}$) as a function of THz frequency under the variation of temperature **(a)** real part and **(b)** imaginary part with ${\varepsilon }_{\infty }=15.68$, $\gamma =\pi \times {10}^{11}\text{ rad} {\text{s}}^{-1}$ and ${E}_{g}=0.26\text{ eV}$.

The electromagnetic modelling of the indium antimonide (InSb) has been done in the frame work of hybrid Drude model as^17,19,20^;

18 $${\varepsilon }_{\text{InSb}}={\varepsilon }_{\infty }-\frac{{\omega }_{p}^{2}}{{\omega }^{2}+i\gamma \omega },$$

where ${\omega }_{p}$ is plasma frequency, ${\omega }_{p}=\sqrt{N{q}_{e}^{2}/0.015{\varepsilon }_{0}{m}_{e}}$, ${q}_{e}=-1.60\times {10}^{-19}C$, ${m}_{e}=9.11\times {10}^{31}$ kg the high-frequency relative permittivity ${\varepsilon }_{\infty }=15.68$ and damping constant $\gamma =\pi \times {10}^{11} {\text{rad}} {\text{s}}^{-1}$. While the temperature dependence of ${\varepsilon }_{\text{InSb}}$ incorporated via the intrinsic career density relation i.e., $N=5.76\times {10}^{20} \, {T }^\frac{3}{2}\text{ exp}\left(-\frac{{E}_{g}}{2{K}_{B}T}\right)$ where the Eg is the band gap of value ${E}_{g}=0.26\text{ eV}$ and ${K}_{B}$ is Boltzmann constant, ${K}_{B}=8.62\times {10}^{-5}\text{ eV }{\text{K}}^{-1}$. To verify that temperature sensitivity of the InSb, the relative permittivity (${\varepsilon }_{\text{InSb}}$) as function of terahertz frequency under different temperature range $T\in [\text{200 K, 220 K, 240 K, 260 K, 280 K, 300 K}]$ has been presented in Fig. 2.

It is obvious from the Fig. 2 that the permittivity of the InSb highly depends upon the operating frequency and external temperature. The real part and imaginary part both can be tuned by changing the temperature as given in the Fig. 2a,b respectively. In Fig. 2a, it is clear that the real part of permittivity Re(${\varepsilon }_{\text{InSb}}$) turns into to negative values from the positive values as the temperature increases from 200 to 300 K upto the frequency range 10THz effectively, however for the frequency greater than 10 THz the effect of temperature on the Re(${\varepsilon }_{\text{InSb}}$) becomes insignificant. Meanwhile, on contrary the imaginary part of permittivity Im(${\varepsilon }_{\text{InSb}}$) increases with the increase of temperature upto 8 THz frequency range as provided by Fig. 2b. It is obvious that the temperature dependent permittivity of (${\varepsilon }_{\text{InSb}}$) behave as insulator at $T=200\text{ K}$ while for the higher temperature range i.e., $T> 200\text{ K}$, it behaves as metal, as given in Ref.^34^. Further, the interface of adjacent materials supports surface waves only when the real part of permittivity of such materials should have opposite in signs^22^. From the Fig. 2a it can be hypothesized that the temperature dependent control over the permittivity of InSb may also invokes the control over the propagation characteristic of surface waves supported by InSb interface.

### Electromagnetic surface wave propagation on graphene loaded InSb

The conditions describe above are in favor to propagation of surface wave so we are going to study the existence of surface waves on graphene-coated indium antimonide, this part covers the temperature dependent characteristics of surface waves. The contour plot technique has been implemented in Wolfram Mathematica kernel to find out the possible solutions for the unknown wave propagation constant (β) that satisfy the characteristic equations^35^. All these numerical calculations are taken at different values of temperature and chemical potential graphene. The electromagnetic surface waves are highly sensitive to the state of polarization. The TE-polarized surface wave is very loosely confined at the graphene layer when compared to the TM-polarized surface wave^30,35^. Therefore, in the subsequent results, the dispersion curve, phase velocity (V_p_) effective mode index (N_eff_), penetration depth (δ), propagation length (L_p_) and field profiles of TM-polarized surface waves supported by the graphene-loaded indium antimonide InSb structures, have been discussed.

First of all, to study the collective response of THz waves at the interface of graphene–InSb, the dispersion curve analysis has been computed numerically between the angular frequency (ω) and propagation constant Re(β). The dispersion relation has significant role in analyzing the propagation characteristics and nature of wave propagation in media^6^.

**Figure 3:**
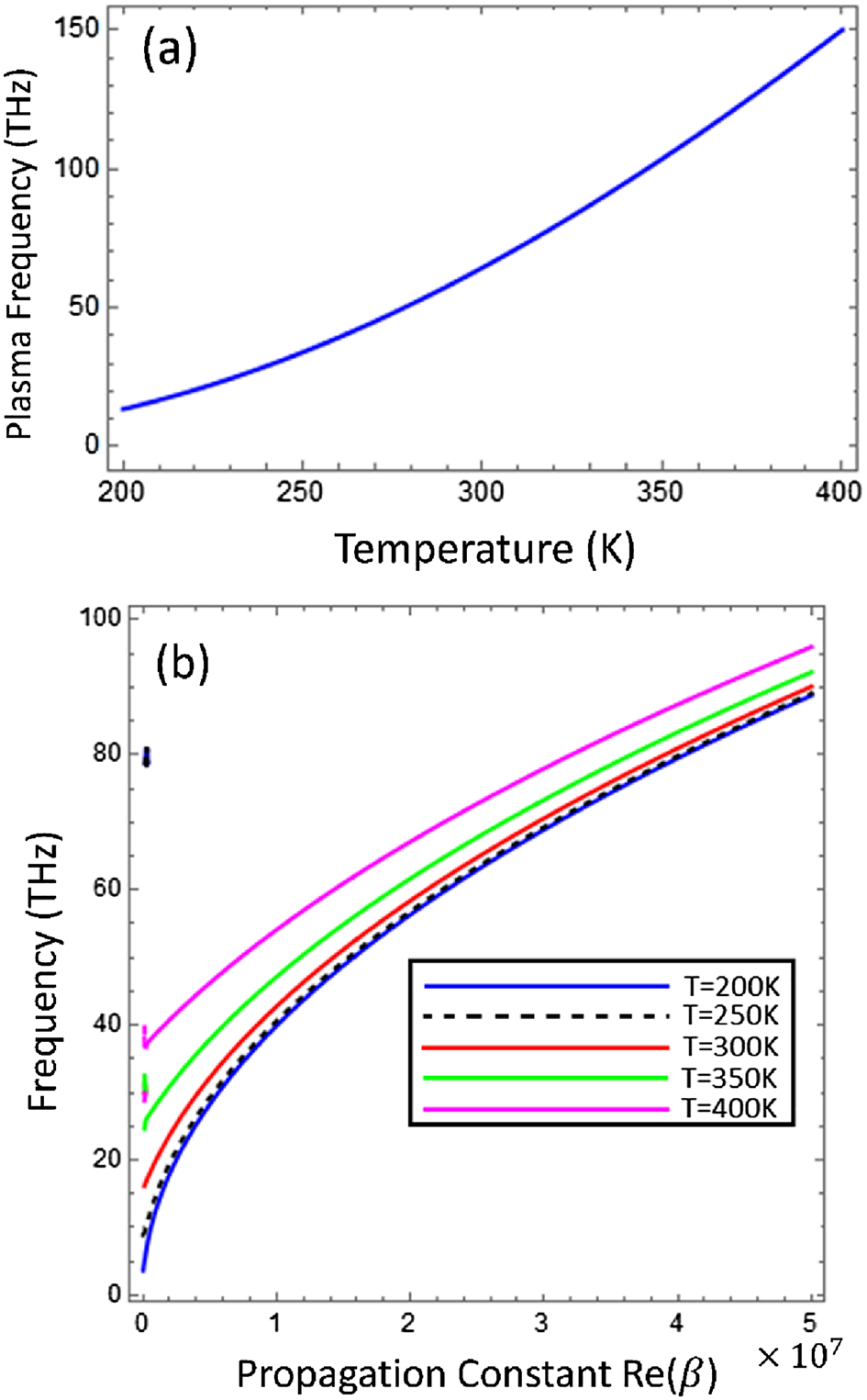
(**a**) Plasma frequency of indium antimonide (InSb) as function of external temperature (T). (**b**) Dispersion curve analysis of TM-Polarized surface waves supported by Graphene-coated indium antimonide under temperature variation with ${\mu }_{c}=0.2\text{ eV}$, $\tau =0.6\text{ ps}$, ${\varepsilon }_{\infty }=15.68$, $\gamma =\pi \times {10}^{11}\text{ rad }{\text{s}}^{-1}$ and ${E}_{g}=0.26\text{ eV}$.

The Fig. 3a depicts the behavior of plasma frequency (${\omega }_{p}$) of indium antimonide as function of temperature (T) and it is clear from the fig that the wit the increase of the temperature the plasma frequency increases and dispersion analysis of the TM-polarized surface waves under the different values of temperature $T\in [\text{200 K}\text{, 250}\text{ K, 300 K, 350 K, 400 K}]$ has been presented in Fig. 3b. It obvious from the Fig. 3 that the temperature as external factor, can be used to tune the propagation characteristics of surface supported by the graphene loaded InSb interface. Further, the dispersion curve presented in Fig. 3b is akin to the dispersion relation of surface plasmon polaritons (SPPs) wave supported by the monolayer graphene as reported in Ref.^33^. For lower temperature range $T\in [\text{200 K}\text{, 250}\text{ K}]$, the impact of temperature on the dispersion curve is minimal because the surface plasmon polaritons of graphene dominate as compared to the InSb, because InSb behaves as dielectric material in this temperature range as provided in Fig. 2. However, for the temperature range $T\in [{300}\text{ K}\text{, 400}\text{ K}]$, the Fig. 3b shows the significant impact of temperature variation on the dispersion curve i.e., the resonance frequency increases with the increase of temperature. In this temperature range, the InSb behaves as conducting material, and plasmons of InSb and graphene coupled and reinforced each other as result the resonance frequency increases.

**Figure 4:**
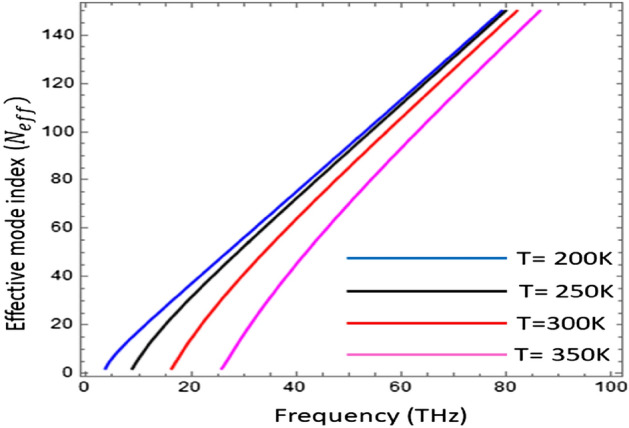
Effective mode index (N_eff_) as a function of frequency under temperature variation with ${\mu }_{c}=0.2\text{ eV}$, $\tau =0.6\text{ ps}$, ${\varepsilon }_{\infty }=15.68$, $\gamma =\pi \times {10}^{11}\text{ rad }{\text{s}}^{-1}$ and ${E}_{g}=0.26\text{ eV}$.

**Figure 5:**
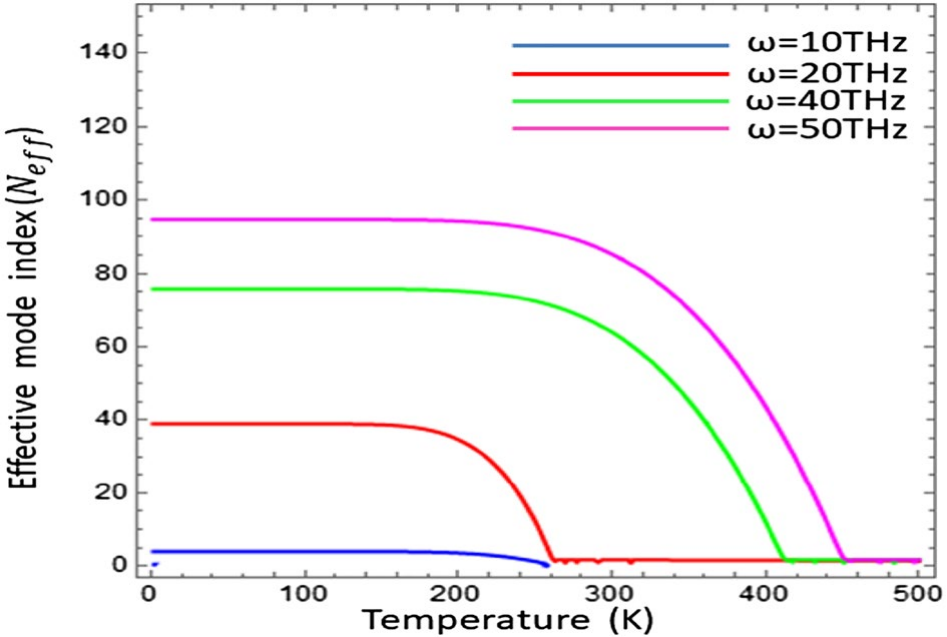
Effective mode index (N_eff_) as a function of temperature under the variation of operating frequency with ${\mu }_{c}=0.2\text{ eV}$, $\tau =0.6\text{ ps}$, ${\varepsilon }_{\infty }=15.68$, $\gamma =\pi \times {10}^{11}\text{ rad }{\text{s}}^{-1}$ and ${E}_{g}=0.26\text{ eV}$.

**Figure 6:**
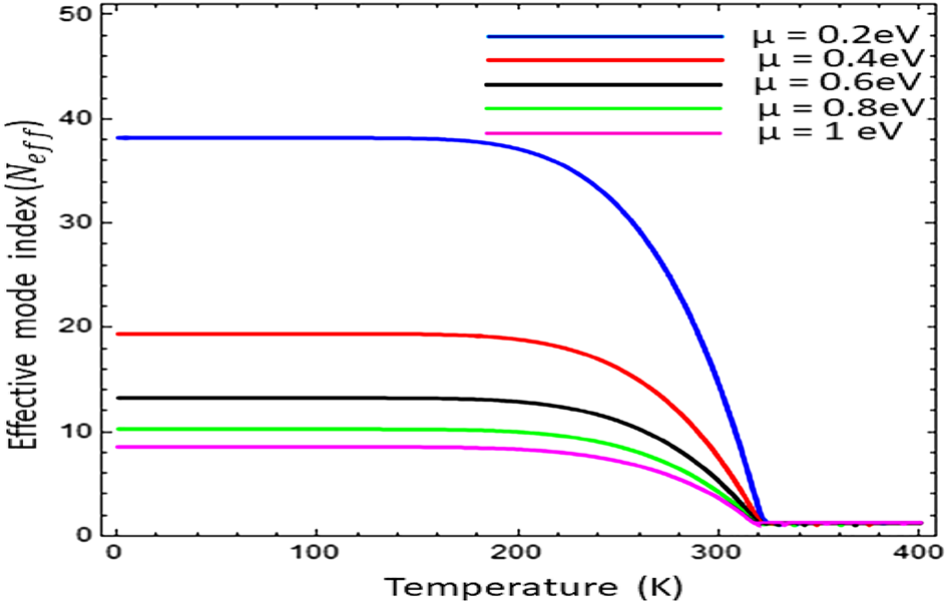
Effective mode index (N_eff_) as a function of temperature under variation of chemical potential ${\mu }_{c}$ with $\tau =0.6\text{ ps}$, ${\varepsilon }_{\infty }=15.68$, $\gamma =\pi \times {10}^{11}\text{ rad }{\text{s}}^{-1}$ & ${E}_{g}=0.26\text{ eV}$.

In Fig. 4 the effective mode index (N_eff_) as function of frequency at different value of temperature i.e., $T\in [\text{200 K}\text{, 250}\text{ K, 300 K, 350 K}]$ has been presented. The effective mode index is calculated as ratio between propagation constant in medium to wave number of free space i.e., ${N}_{eff}=Re(\beta )/{k}_{0}$^35^. The confinement of the surface waves on the interface is estimated by the effective mode index. It is obvious from the figure that the confinement of the surface waves on the graphene–InSb interface is sensitive to the external temperature i.e., with the increase of temperature, the effective mode index is increasing. To further study the impact of effective mode index as function of temperature under variation of operating frequency and chemical potential has been presented in Figs. 5 and 6 respectively.

It is clear from the fig that the effective mode index has the explicitly depends upon the temperature. Moreover, the confinement of the surface wave can be dually controlled by temperature as well as operating frequency. The Fig. 5 shows that the confinement of surface waves corresponds to specific temperature range, which can be further tuned by the operating frequency. The effective mode index increases with the increase of operating frequency $\omega \in [\text{10 THz}\text{, 20}\text{ THz, 40 THz, 50 THz}]$.

**Figure 7:**
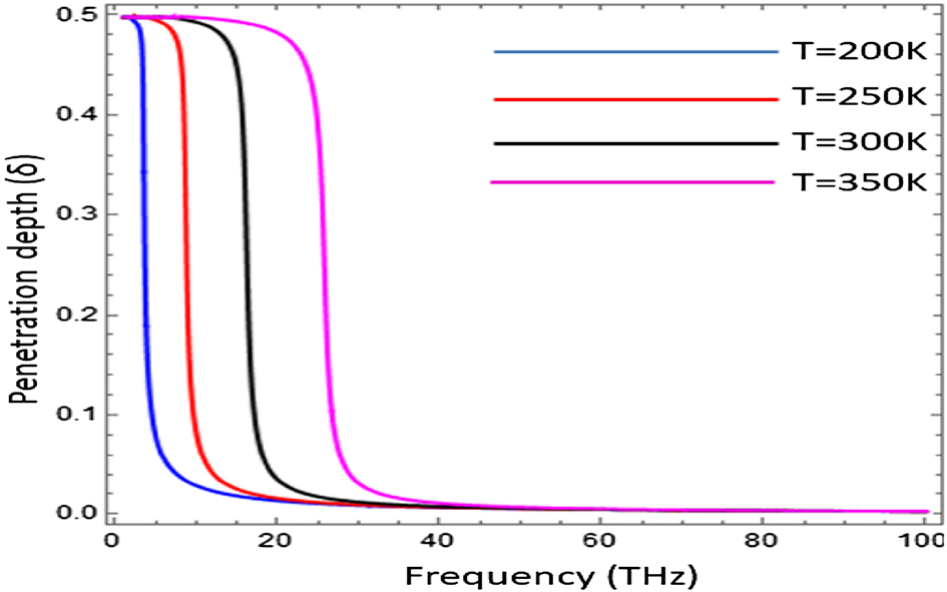
Influence of temperature on the penetration depth $(\delta )$ of surface wave as a function of frequency with ${\mu }_{c}=0.2\text{ eV}$, $\tau =0.6\text{ ps}$, ${\varepsilon }_{\infty }=15.68$, $\gamma =\pi \times {10}^{11}\text{ rad }{\text{s}}^{-1}$ and ${E}_{g}=0.26\text{ eV}$.

**Figure 8:**
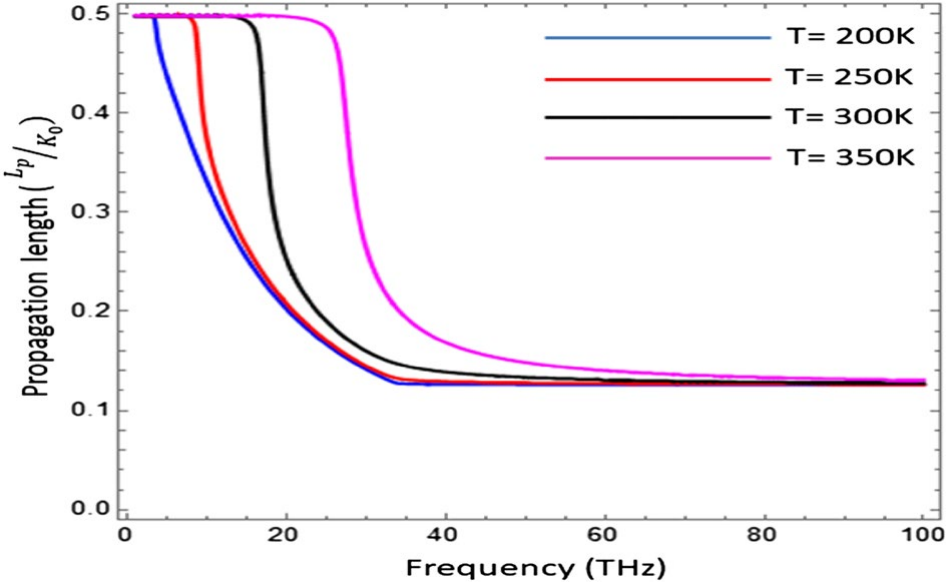
Impact of temperature on propagation length (L_p_) as a function of frequency for the propagation of EM surface waves on the Graphene-based InSb, with ${\mu }_{c}=0.2\text{ eV}$, $\tau =0.6\text{ eV}$, ${\varepsilon }_{\infty }=15.68$, $\gamma =\pi \times {10}^{11}\text{ rad }{\text{s}}^{-1}$ and ${E}_{g}=0.26\text{ eV}$.

The influence of chemical potential $( {\mu }_{c} )$ on effective mode index (N_eff_) as a function of temperature has been presented in Fig. 6. The confinement of electromagnetic surface wave has been analyzed for different value of chemical potential i.e., ${\mu }_{c}\in [\text{0.2}\text{ eV}\text{, 0.4 eV}\text{, 0.6}\text{ eV, 0.8 eV, 1.0 eV}]$. The result shows that with the increase of chemical potential (${\mu }_{c})$ the effective mode index (N_eff_) decreases, but the all the graphs terminated on the same temperature i.e., 320 K. For the temperature $T\ge 320\text{ K}$, the graphene loaded InSb interface does not support the surface as conditions provide in Fig. 6. The penetration depth $(\delta )$ is the measure of penetration of a wave in the medium while propagating on its surface and computed here as $\delta =2\beta /{k}_{0}$^33^. The penetration depth is normalized by wave number of free space i.e., ${k}_{0}=2\pi /{\lambda }_{o}$. The influence of temperature on the penetration depth $(\delta )$ as a function of frequency for surface wave propagation on Graphene-based indium antimonide has been analyzed in Fig. 7. It is clear from the fig that the under-temperature variation $T\in [\text{200 K}\text{, 250}\text{ K, 300 K, 350 K}]$ the normalized pentation depth shows the thermal hysteresis type trend as discussed in Ref.^22^ and with the increase of temperature the hysteresis width increases. In Fig. 8, the dependence of temperature on the normalized propagation length (L_p_) as a function of frequency for the propagation of EM surface waves on the Graphene-based InSb has been presented. The fig shows that the propagation length (L_p_) follows the thermal hysteresis type trend under different values of temperature i.e., $T\in [\text{200 K}\text{, 250 K, 300K, 350 K}]$. For the temperature values i.e., 200 K and 250 K, the propagation length (L_p_) is almost same for higher values of frequency but for temperature range $T\in [\text{300 K}\text{, 350}\text{ K}]$ the propagation length increases with the increase of temperature.

**Figure 9:**
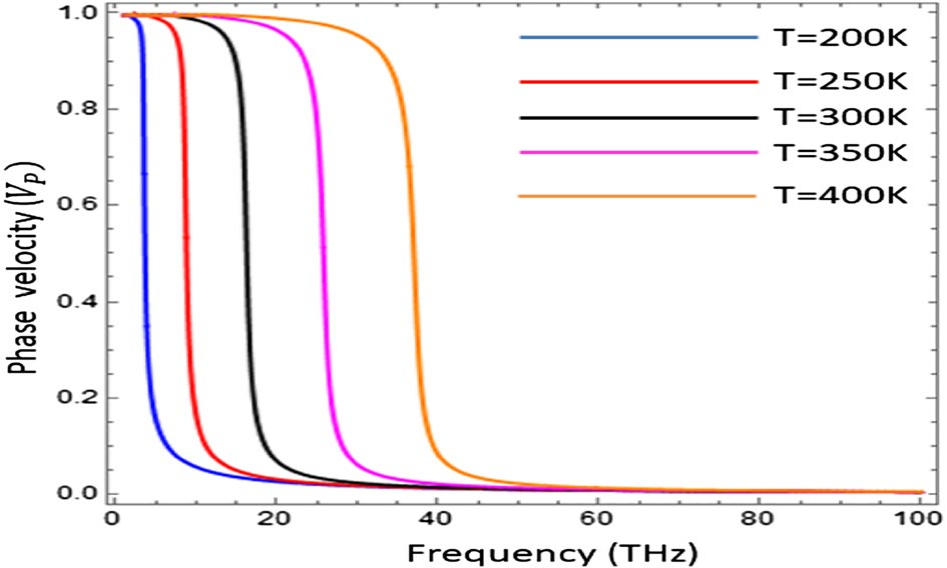
Normalized phase speed (v_p_) as a function of frequency under temperature variation ${\mu }_{c}=0.2\text{ eV}$, $\tau =0.6\text{ ps}$, ${\varepsilon }_{\infty }=15.68$, $\gamma =\pi \times {10}^{11}\text{ rad }{\text{s}}^{-1}$ and ${E}_{g}=0.26\text{ eV}$.

Figure 9 presents the influence of temperature on the phase speed (v_p_) of the surface waves guided by the graphene–InSb interface. To compute the normalized phase speed in kernel, the mathematical definition is used as ${V}_{p}={k}_{0}/Re(\beta )$ and analyzed for the different values of temperature $T\in \left[200, 250, 300, 350, 400\right]\text{ K}$. The normalization of the phase speed (v_p_) has been done with the speed of light. It is clear from the graph that phase speed of electromagnetic surface wave follows the hysteresis trend which can be controlled by tuning the temperature as discussed in Ref.^21^.

**Figure 10:**
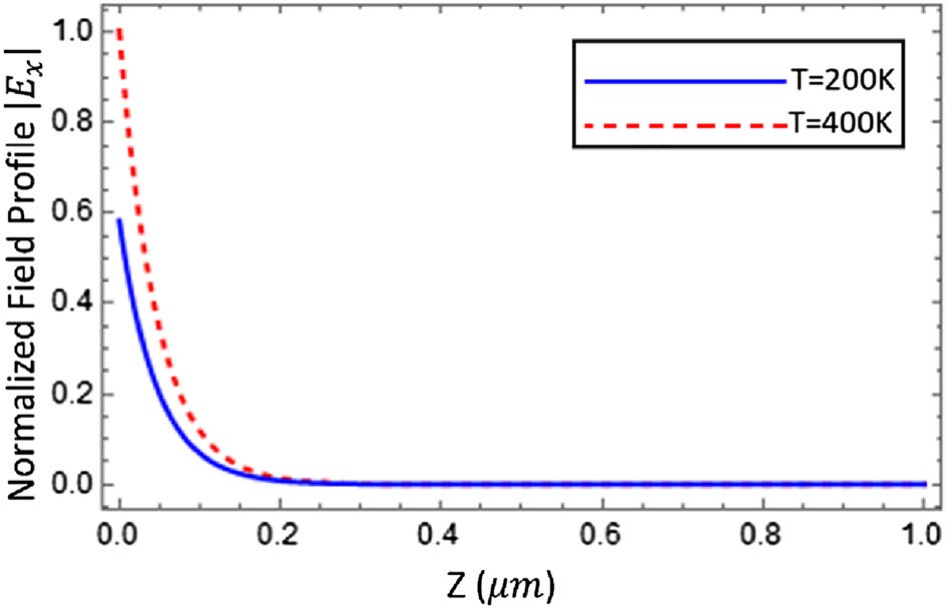
Field profile of electromagnetic surface wave guided by the graphene loaded InSb interface for $T=200\text{ K}$ with ($\omega =58.84\times {10}^{12}\text{ Hz},\beta =2.139\times {10}^{7}{\text{ m}}^{-1})$, and for T = 400 K, with ($\omega =58.24\times {10}^{12}\text{ Hz}, \beta =1.29\times {10}^{7}{\text{m}}^{-1})$, the other physical parameters taken as ${\mu }_{c}=0.2\text{ eV}$, $\tau =0.6\text{ ps}$, ${\varepsilon }_{\infty }=15.68$, $\gamma =\pi \times {10}^{11}\text{ rad }{\text{s}}^{-1}$ and ${E}_{g}=0.26\text{ eV}$.

The field profiles of electromagnetic surface waves supported by Graphene loaded indium antimonide, as a function of transverse distance from interface (z) under different values of temperature i.e., $\mathrm{T}=200\text{ K}$ and $\mathrm{T}=400\text{ K}$, has been presented in Fig. 10. It can be seen that as the distance of curve increases from the interface then the field profile tends to decrease exponentially, which shows that this is a surface wave. Moreover, it has been confirmed that the field distribution highly depends upon the external temperature.

## Concluding remarks

The thermally tunable electromagnetic surface waves supported by the graphene-loaded InSb has been studied. The propagation characteristics of surface waves, such as dispersion curve, effective mode index, penetration depth, normalized phase velocity, propagation length and field profile for the graphene-loaded InSb, are found sensitivities to external temperature and chemical potential. The following conclusions have been drawn from the numerically computed results:

(i) The electromagnetic surface waves guided by the graphene-loaded indium antimonide (InSb) interface are akin to the to the surface plasmons polaritons (SPPs) waves.
(ii) The temperature as an external parameter plays an active role in controlling the propagation characteristics of surface waves i.e., dispersion relation, confinement of surface waves and phase speed, penetration depth, propagation length and field profile.
(iii) The chemical potential/doping of graphene also provide additional degree of freedom to tune and modify the propagation characteristics of the surface waves.
(iv) The numerical results for penetration depth, propagation length and phase speed reveal that they have thermal hysteresis type dependence against temperature and frequency variation.
(v) The computed numerical results in this work may have potential applications in thermo-optical sensor designing, THz photoemission temperature assisted communication devices, near field thermal imaging and spectroscopy platforms designs.

## Data Availability

All data generated or analyzed during this study are included in this published article.
